# Expression and Purification of ADAMTSL5 in *E. coli* and Validation of Activity in Psoriasis Serum

**DOI:** 10.1002/iid3.70100

**Published:** 2024-12-13

**Authors:** Ru Qin, Shangyang Li, Boheng Wu, Ruilan Lin, Yulin Yuan

**Affiliations:** ^1^ Department of Clinical Medicine Guilin Medical University Guilin Guangxi China; ^2^ Department of Laboratory Medicine The People's Hospital of Guangxi Zhuang Autonomous Region,The Guangxi Key Laboratory of intelligent precision medicine Nanning Guangxi Zhuang Autonomous Region China

**Keywords:** ADAMTSL5, expression, psoriasis, purification

## Abstract

**Purpose:**

A disintegrin and metalloprotease domain containing thrombospondin type 1 motif‐like 5 (ADAMTSL5), a protein linked to psoriasis, was obtained by prokaryotic expression and purification for potential utilization as a new auxiliary diagnostic marker for psoriasis. It was subsequently applied in psoriasis research.

**Experimental Design:**

The designed ADAMTSL5 gene was inserted into the pET‐30a (+) vector and expressed in the BL21 (DE3) strain as a fusion protein. Following this, the recombinant ADAMTSL5 protein was purified using affinity chromatography. Purified ADAMTSL5, obtained in conjunction with magnetic beads, was directly employed in both psoriasis patients and healthy individuals.

**Results:**

The results indicated that the molecular weight of the ADAMTSL5 protein obtained through prokaryotic expression and purification was approximately 27 kDa, with a protein concentration of 0.96 mg/mL. Analysis of anti‐ADAMTSL5 levels in the serum of both psoriasis patients and healthy individuals using magnetic microparticle chemiluminescence demonstrated that anti‐ADAMTSL5 levels in the serum of psoriasis patients surpassed those of healthy individuals, showing a significant difference with *p* < 0.001.

**Conclusions:**

The experimental findings presented here may contribute to the utilization of ADAMTSL5 as a marker for diagnosing psoriasis, offering novel insights and experimental avenues for further investigation.

## 1 Introduction

In recent years, ADAMTSL5, a newly discovered protein, has emerged as an activated antigen of psoriasis. Its association with ADAMTS metalloproteinases is due to its resemblance to the ADAMTS auxiliary domain [1]. Despite this, ADAMTSL5 stands out as a new member within the ADAMTS superfamily, characterized by a distinct modular organization featuring a singular C‐terminal netrin‐like (NTR) module [2]. At its 5′end, alternative splicing of ADAMTSL5 yields two transcripts encoding distinct signal peptides but the same mature protein, showing variations in their translation efficiencies [3]. The recombinant form of ADAMTSL5, a secreted N‐glycosylated 60 kDa glycoprotein, is situated within the subcellular matrix, on the cell surface, and in the media of transfected cells. In their study, Hannah L. et al. demonstrated that ADAMTSL5 binds to recombinant fibrillin‐1 and fibrillin‐2, colocalizing with fibrin microfibrils in the extracellular matrix of cultured fibroblasts, albeit without an apparent impact on microfibril assembly [4]. Notably, this protein represents the first within its family to exhibit binding affinity to fibrillin‐1 and fibrillin‐2. Much like its counterpart in the ADAMTS family, ADAMTSL5 likely plays a role in regulating microfiber function, recognizing both human and animal mutations. The involvement of ADAMTSL5 in microfibril formation garners significant attention as a pivotal mechanism in growth factor regulation within the extracellular matrix [4].

Psoriasis, a chronic skin disease mediated by the immune system, is characterized by skin erythema and scales, and presents a protracted course prone to relapse, with certain patients enduring symptoms throughout their lives. The condition manifests through excessive proliferation and abnormal differentiation, clinically resulting in variously sized erythematous squamous plaques. Initially, psoriasis was misinterpreted as a leprosy variant until von Hebra reclassified it as a distinct disease [5]. Affected individuals typically exhibit well‐defined chronic erythematous plaques adorned with silver‐white scales, predominantly localized in areas such as the knee, elbow, scalp, umbilical region, and lumbar areas [6]. Psoriasis, a genetically intricate condition, arises from diverse risk factors instigating multiple processes encompassing inflammation, antigen presentation, cell signaling, and transcriptional regulation [7], with a T cell‐mediated immune response implicated as a causative factor. Notably, the psoriasis susceptibility locus 1 (PSORS1), primarily associated with the human leukocyte antigen C, confers the highest risk [8]. More than 60% of patients carrying the allele human leukocyte antigen C * 06:02, exhibit a heightened predisposition, particularly C * 06:02 homozygotes displaying a five‐fold increased risk compared to heterozygotes [9, 10]. Arakawa [11] described that ADAMTSL5 may serve as an activated antigen in the T cell activation pathway, particularly when presented by the high‐risk allele HLA‐C * 06:02. Moreover, ADAMTSL5 expression extends beyond melanocytes to encompass keratinocytes and perivascular dermal cells [12], while T cells within psoriatic skin lesions predominantly inhabit the epidermis. The discernible oligoclonality of the T cell population in psoriatic skin lesions indicates localized antigen presentation drives psoriatic T cell activation [13, 14, 15, 16, 17]. To enhance understanding of the relationship between ADAMTSL5 and psoriasis, this study devised a cost‐effective bioengineering technique to produce recombinant ADAMTSL5 by incorporating ADAMTSL5 into *E. coli*, facilitating protein expression and purification. Utilizing purified protein, researchers assessed the level of anti‐ADAMTSL5 in human serum. This prokaryotic expression system, yielding ample quantities, coupled with its mature technology, establishes a framework for subsequent ADAMTSL5 functional studies and the auxiliary diagnostic utility in psoriasis.

## 2 Materials and Methods

### 2.1 Sample Collection

From June 2023 to December 2023, 30 patients diagnosed with psoriasis were selected consecutively from the Department of Dermatology and Venereal Diseases at the People's Hospital of Guangxi Zhuang Autonomous Region, along with 30 healthy donors from the health examination center. Patients with psoriasis including 23 males and 7 females aged 24–80 years, with an average age of 48 ± 13 years. Age of the healthy donors ranged from 23 to 58 years, and the average age was 39 ± 8 years. Patients with psoriasis were omitted if they had chronic inflammatory, allergic, autoimmune, metabolic, neoplastic disease, or any condition that might influence psoriasis results. Venous blood (3–5 mL) was collected using disposable blood collection needles into centrifuge tubes, which were then centrifuged at 3000 rpm for 10 min. Following centrifugation, the upper serum was extracted and stored in a −80°C refrigerator.

### 2.2 Materials

GeneScript Biotech Crop synthesized the cDNA of ADAMTSL5 (Genbank number: BC139833.1). Invitrogen Corporation provided plasmid pET‐30a (+). Genstar Inc. supplied Bam HI enzyme and EcoRI. Professor Yuan Yulin from the People's Hospital of Guangxi Zhuang Autonomous Region provided the BL21 (DE 3) strain. Novozymes Corporation supplied dNTPS, Taq DNA polymerase, and Pyrobest high‐fidelity DNA polymerase. Beijing Auboxing Biotechnology Co. Ltd. provided LB nutrient agar medium and LB broth medium. The FastPure Gel DNA Extraction Mini Kit was procured from Nanjing Vazyme Biotech Company. Beijing Quanshijin Biotechnology Co. Ltd. Supplied the plasmid extraction kit. Shanghai Wansheng Haotian Biotechnology Co. Ltd. Provided GLASS gel and Hepes‐Tris. LodeStars supplied magnetic bead particles, while Agilent provided Carboxyl magnetic beads. Secondary antibodies were obtained from Mouse anti‐human IgG5E9 from Qingdao Shuojing Biotechnology Co. Ltd.

### 2.3 Method

#### 2.3.1 Construction and Characterization of the ADAMTSL5‐pET‐30a Vector

**Figure 1:**
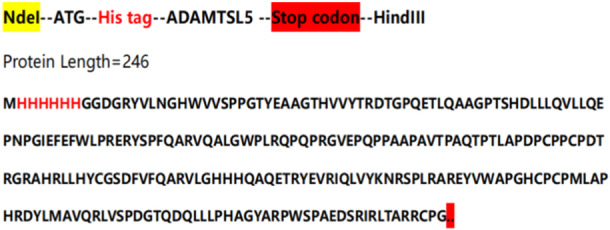
ADAMTSL5 gene sequence.

The fusion gene sequence of ATAMSTL5 was arranged in the following manner: NdeI‐ATG‐His tag‐ADAMTSL5‐Stop codon‐HindIII. The gene spans 753 base pairs and was cloned into the pET‐30a vector (Figure 1). Primer design included upstream ADAMTSL5‐F: 5′ ‐TGTATACCCGTGACACTGGT‐3′, and downstream ADAMTSL5‐R: 5′ ‐GATCCCGCGAAATTAATACG‐3′. PCR amplification utilized the synthesized ATAMSTL5 gene sequence as a template with ADAMTSL5‐F and ADAMTSL5‐R as primers (preheating at 95°C for 5 min, denaturing at 95°C for 30 s, annealing at 65°C for 30 s, extending at 72°C for 90 s, repeated for 34 cycles). PCR products were subjected to 15% agarose gel electrophoresis, and the ATAMSTL5 gene fragment was recovered using a DNA gel recovery kit. These fragments were then transferred into E. coli DH 5α competent cells and screened in LB medium containing kanamycin resistance. Confirmation of the expression vector was attained by running an agarose gel electrophoresis following double digestion with Bam HI and EcoRI.

#### 2.3.2 IPTG Induced the Engineered Bacteria to Express the ADAMTSL5

To incorporate the plasmid into E. coli competent for ADAMTSL5 expression, the following steps were performed: BL21 (DE3) competent cells were retrieved from an ultra‐cold refrigerator, thawed on ice, and gently mixed with BL21 (DE3). The mixture was then incubated on ice for 30 min, followed by a 90 s heat shock at 42°C in a water bath and a 3 min incubation on ice. Subsequently, 100 µL of room temperature LB liquid medium was added, and the culture was shaken at 200 rpm for 60 min at 37°C. The cultured broth was collected, mixed, and spread onto a kanamycin‐resistant plate medium. The plates were inverted and incubated overnight at 37°C. Overnight kept cultures were extracted, and three monoclonal bacteria were selected and inoculated into 4 ml LB tubes containing 50 µg/mL of kanamycin. The tubes were then incubated at 37°C with shaking at 200 rpm until the OD600 reached 0.6 ~ 0.8. Further, 0.5 mM IPTG was added to two tubes, followed by incubation at 15°C for 16 h and 37°C for 4 h. The last tube served as a negative control. Lastly, the precipitate from 450 µL of the medium was removed, resuspended in 300 µL of lysis buffer (50 mM Tris‐HCl, 500 mM NaCl, 5% glycerol, 0.5% TritonX‐100, pH 8.0), and lysed by sonication for 1 min.

#### 2.3.3 Whole Bacteria Sample Preparation

From the aforementioned steps, 100 µL of lysate was extracted, thoroughly mixed with 50 µL 5x loading buffer, and heated at 100°C for 10 min, followed by centrifugation at 15,000 revolutions for 5 min.

#### 2.3.4 Preparation of Supernatant and Inclusion Body Samples

The remaining 200 µL of lysate underwent centrifugation at 15,000 rpm for 10 min, leading to the separation of the supernatant and precipitate. To prepare supernatant samples, 90 µL 5x loading buffer was added to 180 µL of supernatant. The inclusion body sample was obtained by resuspending the precipitate in 150 µL 5x loading buffer. Following heating at 100°C for 10 min, samples were centrifuged at 15,000 rpm for 5 min. Lastly, the assessment of whole bacteria, supernatant, and solubility was conducted using SDS‐PAGE and Western blot analysis.

#### 2.3.5 Purification of the ADAMTSL5

Affinity chromatography was utilized to purify the ADAMTSL5 protein. Thawed glycerides were inoculated into 4 ml of LB medium containing 50 µg/mL kanamycin and cultured overnight in a shaker at 37°C, 200 rpm. Subsequently, the 4 mL seed solution was transferred into 2500 mL (50 µg/mL kanamycin) TB medium in a 2 L Erlenmeyer flask and incubated at 37°C with shaking at 200 rpm. Once the OD600 of the culture reached 1.2, protein expression was induced by the addition of IPTG to a final concentration of 0.5 mM and further incubated at 15°C for 16 h. Following incubation, the solution was extracted and centrifuged at 4°C, 8000 rpm to collect the resulting precipitate, yielding 30.8 g of bacteria. This precipitate was then resuspended in 150 ml of lysis buffer (50 mM Tris‐HCl, 150 mM NaCl, 1% Triton, 2 mM DTT, pH 7.5, and a small amount of nuclease). Cells underwent lysis utilizing a sonicator (3 s, suspended for 6 s, 6 min, power 600 W), and the resulting cell lysate was centrifuged at 10,000 rpm for 22 min to discard the supernatant. The inclusions were dissolved in 150 mL of denaturing buffer (50 mM Tris‐HCl, 7 M guanidine hydrochloride, 2 mM DTT, pH 8.0). Subsequently, the dissolved inclusion bodies were centrifuged at 10°C, 12,000 rpm for 30 min, and the supernatant was extracted for advanced purification. Five milliliters Ni column was used to purify the targeted proteins in the aforementioned supernatant. The Ni column was pre‐equilibrated with 50 mL binding buffer (50 mM Tris‐HCl, 8 M urea, pH 8.0) as a column equilibration buffer, then loaded and washed with 50 mL of wash buffer (50 mM Tris‐HCl, 8 M urea, 20 mM imidazole, pH 8.0). Lastly, the target protein was eluted with elution buffer (50 mM Tris‐HCl, 8 M urea, 50 mM imidazole, 2 mM DTT, pH 8.0), and 50 mL/tube was collected. The fractions obtained during the purification process were subsequently analyzed using SDS‐PAGE.

Samples eluted with 50 mM imidazole were collected, and SDS was added to achieve an ultimate concentration of 0.2%. The samples were then refolded by dialysis into 1X PBS buffer containing 0.2% SDS at pH 7.4. Dialysis was conducted overnight using a 14.0 kDa truncated dialysis membrane with a dialysis ratio exceeding 1:100. Following dialysis, the samples were filtered through a 0.22 µm filter. Quality tests for the final purification involved SDS‐PAGE and Western blot analysis.

#### 2.3.6 ADAMTSL5 Clinical Validation

To ascertain the relationship between prokaryotically expressed Tao white and psoriasis, purified ADAMTSL5 and magnetic microparticles were coated and incubated with human serum and secondary antibodies. Subsequently, the detection of the content of anti‐ADAMTSL5 antibodies in the serum was carried out by using a chemiluminescence instrument. The content of anti‐ADAMTSL5 antibodies in the serum of healthy individuals and psoriasis patients was compared to assess ADAMTSL5 activity. The specific steps were as follows: A clean centrifuge tube was taken, 100 µL of coated liquid was mixed with 900 µL, and the supernatant was discarded. Subsequently, 900 µL of coated liquid was added to 100 µL of EDC, spun for 30 min, and the supernatant was removed. Lastly, 900 µL of coated solution and 100 ug of ADAMTSL5 protein were combined, sonicated for 20 min, and then centrifuged to discard the supernatant. The mixture was washed three times with 10 mL plus 900 µL blocking solution, followed by another round of sonication for 20 min, and removal of supernatant. Subsequently, the prepared ADAMTSL5 magnetic beads, Mouse anti‐human IgG5E9, and diluent were placed into the reaLmind automatic chemiluminescence analyzer. Thirty sera from healthy individuals and 30 sera from psoriasis patients were consecutively added to the upper sample of the instrument. The luminescence values were recorded, and the data were analyzed using GraphPad Prism software.

## 3 Results

### 3.1 Construction and Characterization of the ADAMTSL5‐pET‐30a Vector

**Figure 2:**
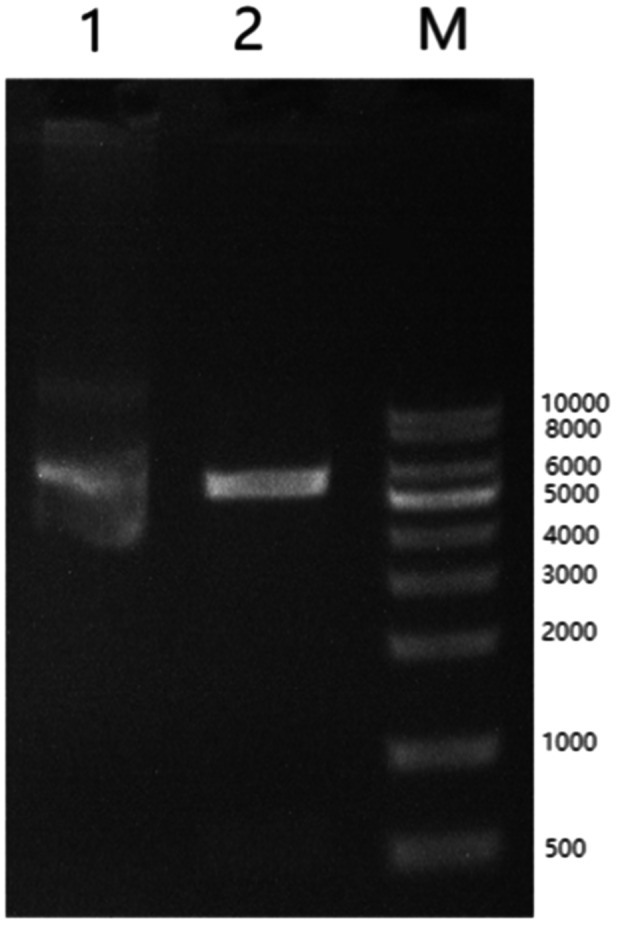
Agarose gel electrophoresis. Lane M: KB Ladder; Lane 1: ADAMTSL5‐pET‐30a (+) plasmid; Lane 2: ADAMTSL5‐pET‐30a (+) plasmid digested by EcoRI and HindIII.

ADAMTSL5 Protein is secreted, so to facilitate its successful expression in prokaryotes, the amino acid sequence of the second half was cleaved (Figure 1), resulting in a protein of length 735 bp. According to Bader's study, ADAMTSL5 exhibits two novel structural features: the lack of a TSR module at the C terminus and the presence of an NTR module [1]. The NTR module comprises six cysteines and typically forms disulfide bonds to the patterns: C1‐C4, C2‐C5, C3‐C6, along with a conserved hydrophobic/basic motif. These residues within the ADAMTSL5NTR module are conserved and possess an akin structure. ADAMTSL5, containing fragments of the NTR module, was retained for expression. Through overlapping PCR reactions, an ADAMTSL5 fusion gene with a fragment length of 753 bp was synthesized. Monoclonal strains acquired from the recombination reaction were then amplified by PCR using primers ADAMTSL5‐F and ADAMTSL5‐R. The target fragment ATAMSTL5 gene sequence was recovered using a DNA gel recovery kit. Subsequently, the expression vector Nde I‐ATG‐His tag‐ADAMTSL5‐Stop codon‐HindIII was transferred into *E. coli* DH 5 α competent cells for screening in LB medium containing kanamycin resistance. Confirmation of the expression vector was conducted through agarose gel electrophoresis subsequent to double digestion with Bam HI and EcoRI (Figure 2).

### 3.2 IPTG Induced Engineered Bacteria to Express the ADAMTSL5

**Figure 3:**
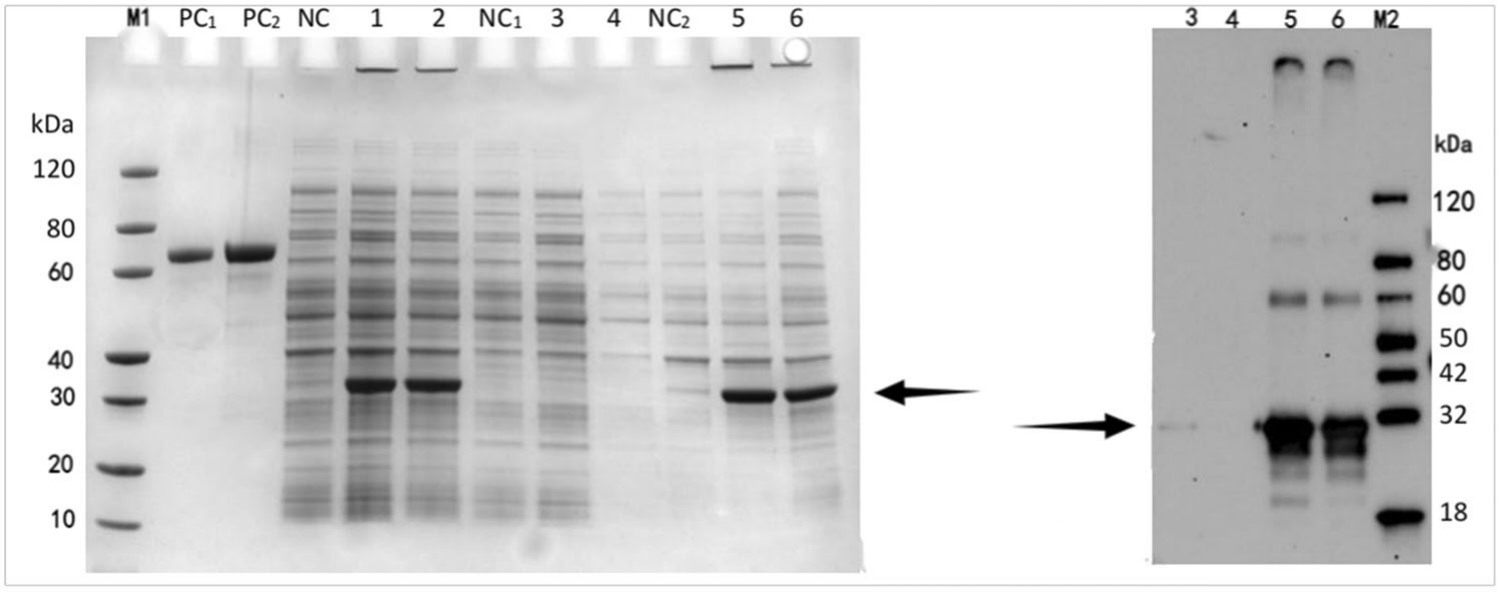
ADAMTSL5 Protein was analyzed by SDS‐PAGE (left) for BL21 (DE3) expression and Western blot (right): Lane M1: Protein marker; Lane M2: Western blot protein marker; Lane PC1: BSA (1 µg); Lane PC2: BSA (2 µg); Lane NC: Uninduced whole cell; Lane 1: Induction at 15°C for 16 h in whole cell; Lane 2: Induction at 37°C for 4 h in whole cells; Lane NC1: Uninduced cell lysis supernatant; Lane 3: Supernatant of the sample induced at 15°C for 16 h; Lane 4: Supernatant of the sample induced at 37°C for 4 h; Lane NC2: Cell lysate of the uninduced sample; Lane 5: 15°C for 16 h; Lane 6: Sample induced at 37°C for 4 h. For the Western blot, an anti‐His antibody was used.

The designed ADAMTSL5 protein's molecular weight in this experiment is nearly 27 kDa. In the engineered bacterium ADAMTSL5‐pET‐30a/BL21 (DE3) induced with 0.5 mM IPTG, the supernatant and precipitate of the bacteria were evaluated through SDS‐PAGE and Western blot, respectively. A distinct band was observed around 30 kDa (Figure 3). The protein's molecular weight coordinated the hypothetical value, and no consistent band was observed deprived of IPTG induction (Figure 3), indicating the effective ADAMTSL5 protein expression. Since the expression region of the ADAMTSL5 protein lacks a signal but may not form a disulfide bond, and inclusion bodies are expected to form, the experimental outcomes also confirmed that ADAMTSL5 protein primarily occurs in the precipitate, signifying insoluble expression of the recombinant protein ADAMTSL5 in BL21 (DE 3). The optimum induction condition for ADAMTSL5 was 16 h at 15°C, resulting in an expression level of 20 mg/mL and a soluble ratio of less than 1%.

### 3.3 Purification of the ADAMTSL5

**Figure 4:**
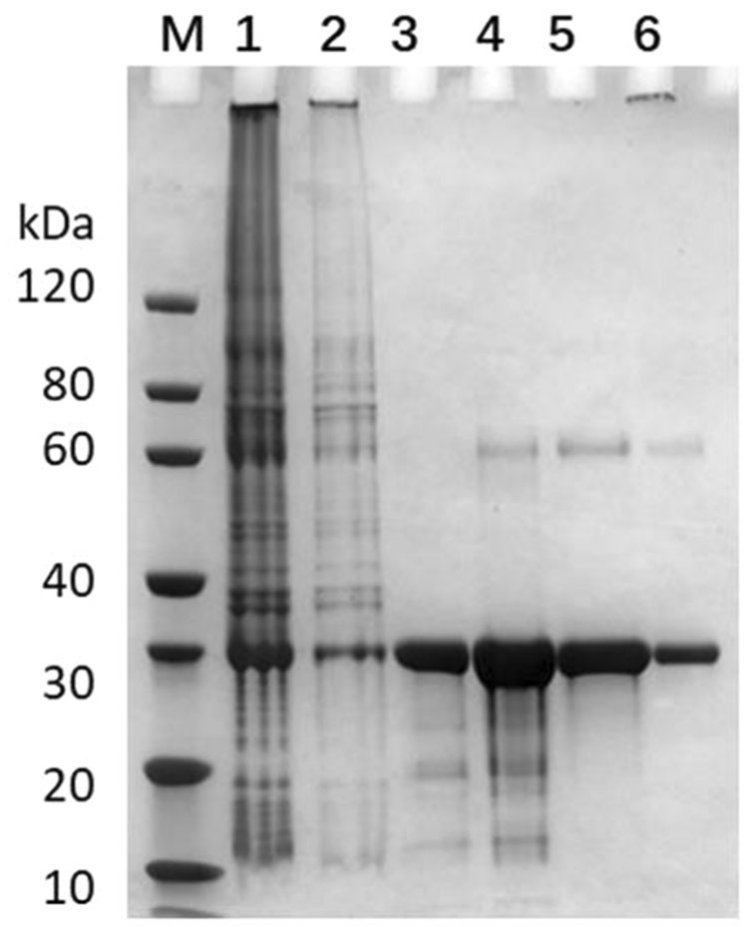
SDS‐PAGE analysis of the ADAMTSL5 protein purified from BL21 (DE 3) cells. Lane M: Protein marker; Lane 1: Loaded sample; Lane 2: Flowthrough fraction; Lane 3:20 mM imidazole eluted sample; Lane 4:50 mM imidazole eluted sample; Lane 5:500 mM imidazole eluted sample; Lane 6: Residue left on the column.

**Figure 5:**
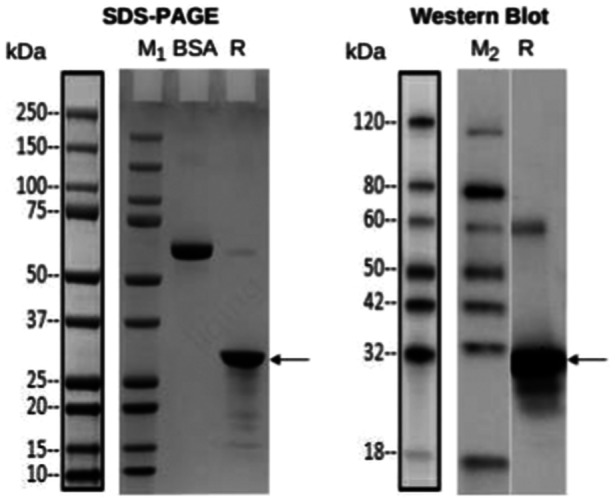
SDS‐PAGE & Western blot Analysis: LaneM1: Protein Marker, Marker band size according to the left template; LaneM2: protein Marker, Marker band size according to the left template; BSA: 2.00 pg; R: reducing condition; antibody: Mouse‐anti‐His mAb.

The supernatant and precipitate of the bacteria underwent elution via affinity chromatography. The target protein was subsequently eluted with 50 mM imidazole in an elution buffer (Figure 4), and the eluate was collected for SDS‐PAGE and Western Blot analysis using Mouse‐anti‐His mAb (Figure 5). As depicted in Figure 5, a distinct band emerged near 30 kDa, with the arrow specifying the purified ADAMTSL5 protein, confirming successful purification.

### 3.4 ADAMTSL5 Clinical Validation

**Figure 6:**
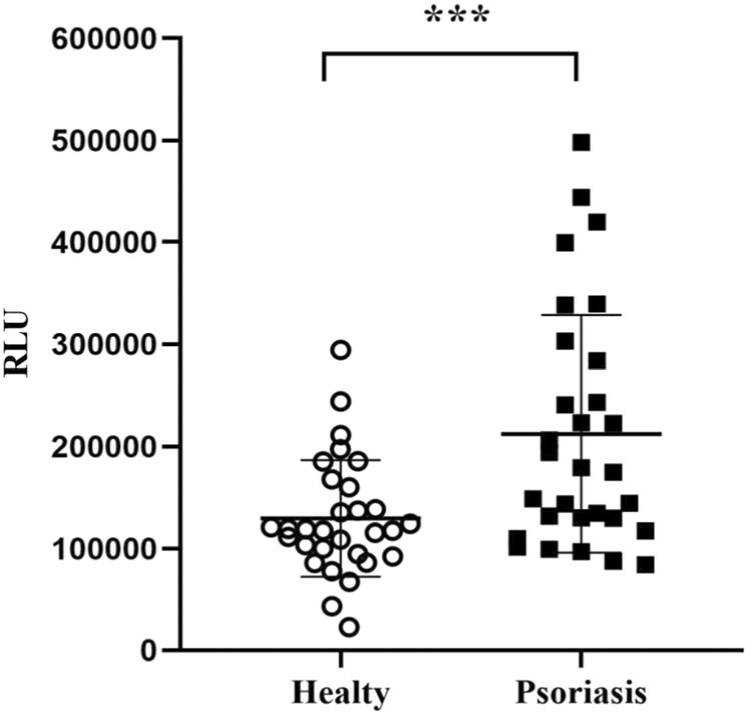
For ADAMTSL5 coated magnetic beads at a concentration of 10 µg/mL and Mouse anti‐human IgG5E9 at 0.05 mg/mL, analyzed using the T‐test in GraphPad software. The RLU values of anti‐ADAMTSL5 in the serum of healthy people and psoriasis patients were significantly different, ***p < 0.001.

The independent sample *t*‐test analysis of luminescence value data performed using GraphPad Prism software indicated that the anti‐ADAMTSL5 luminescence level in the serum of psoriasis patients was higher than that in healthy individuals. The anti‐ADAMTSL5 levels ranged from 22,886 to 294,136 RLU, with an average of 129,000 ± 56,341 RLU in psoriasis patients. For healthy donors, anti‐ADAMTSL5 levels ranged from 84,285 to 498,096 RLU, with an average of 212,326 ± 114,494 RLU. According to the formula in Table 1, the sample size rationality was calculated to be *n* = 10 cases. Considering a 1:1 randomization into intervention and control groups, each group required 10 cases. Accounting for a 15% follow‐up loss and refusals, 12 cases per group were deemed necessary, totaling at least 24 cases. Thus, a sample size of 30 cases per group would be sufficient to demonstrate the effectiveness of the experimental results. Additionally, a notable disparity was observed between the levels of anti‐ADAMTSL5 in the serum of healthy individuals and those with psoriasis *** *p* < 0.001 (Figure 6). These findings align with those reported by Yulin Yuan et al. [18] demonstrating that the ADAMTSL5 protein expressed by *E. coli* can indeed bind to anti‐ADAMTSL5 in psoriasis. To provide a clearer view of the ADAMTSL5 expression level in psoriasis patients, only the anti‐ADAMTSL5 level was analyzed, without employing additional methods. This single assay approach may not ensure the efficient specificity and accuracy of the ADAMTSL5 protein, which represents a limitation of this experiment.

**Table 1:** The formula for sample size calculations is as follows: *n* represents the sample size; α denotes the significance level; β is the control value; σ indicates the standard deviation of the control group; and δ is the difference between the means of the two groups.

$n=\frac{2{\left(z_{\alpha }+z_{\beta }\right)}^{2}*\sigma^{2}}{\delta^{2}}$

## 4 Discussion

This study confirmed the expression vector, ADAMTSL5‐pET‐30a, which was designed to overproduce a protein containing ADAMTSL5. The ADAMTSL5 gene was introduced into the BamHI and EcoRI restriction sites within the multiple cloning sites of pET‐30a. Confirmation of the expression vector was achieved through electrophoresis (Figure 2), and ADAMTSL5 expression was validated (Figure 3). Subsequently, ADAMTSL5 was purified (Figures 4 and 5), with a protein concentration of 0.96 mg/mL. The biological activity of ADAMTSL5 was evaluated by determining the anti‐ADAMTSL5 level using magnetic beads. According to Favaro [19]. The autoantigens LL37 and ADAMTSL5 contribute to induce pathogenetic T‐cell responses in a subset of psoriatic patients. As this experiment aimed solely to investigate the relationship between ADAMTSL5 and psoriasis and to evaluate the potential of ADAMTSL5 as a novel psoriasis marker, reactive T cells were not assessed. The detection method will be refined in subsequent research. Nevertheless, this experiment offers technical support for the production of ADAMTSL5 and contributes scientific value to understanding the relationship between ADAMTSL5 and psoriasis. It explores the potential of ADAMTSL5 as a new psoriasis marker, which may serve as a reference for differential diagnosis.

Despite the utilization of commercially procured recombinant proteins in some studies, there were no reports available on the recombinant expression of ADAMTSL5 in the *E. coli* expression system. Eukaryotic systems can express proteins with proper folding and posttranslational modification, enabling the expression of complex antibody structures and facilitating antibody secretion for easy extraction and purification. Nevertheless, eukaryotic expression systems have drawbacks, including expensive intricate manipulation and comparatively minimal expression levels. Since ADAMTSL5 is a novel protein identified in this century, there is limited research data available on it. Consequently, the present study investigates the prokaryotic expression system for ADAMTSL5 purification, considering its simplicity, cost‐effectiveness, and high expression levels. Producing large quantities of ADAMTSL5 at a low cost is crucial for facilitating subsequent mechanistic studies of it and the disorder.

ADAMTSL5 serves as both an activated antigen for CD8 + IL‐17 T cells in psoriasis and, as demonstrated in Arechederra et al.'s study [20], plays a role in sustaining vital oncogenic signaling pathways, indicating its pivotal role in tumorigenesis and resistance in HCC. The expression of ADAMTSL5 in this investigation offers essential material for in‐depth exploration of its involvement in elucidating the pathogenesis of psoriasis and other diseases. According to Prinz [21], autoimmune responses specific to melanocytes, within the framework of immune mechanisms linked to HLA function and T‐cell receptor‐antigen recognition, have significant inferences for the expression, regulation, and therapeutic modulation of psoriatic autoantigens. Overall, numerous studies [9, 10, 11, 12] have highlighted the adjacent association between ADAMTSL5 and psoriasis. To deepen the understanding of the connection between ADAMTSL5 and psoriasis, this study employs prokaryotic expression and purification methods to generate ADAMTSL5; thus, providing raw material and production technology for consequent psoriasis research. Additionally, chemiluminescence was used to validate ADAMTSL5, introducing a novel approach as no prior literature or technology has addressed the combination of ADAMTSL5 and magnetic microparticle chemiluminescence technology, therefore offering a fresh perspective for ADAMTSL5 verification.

## 5 Conclusion

ADAMTSL5, a recently discovered protein, is currently under investigation for its immune mechanism and its association with disease, rendering it a highly promising subject of study. To effectively conduct research, obtaining ample quantities of reasonable ADAMTSL5 necessitates the use of recombinant techniques. While the eukaryotic expression system presents challenges such as intricate, significant expenses and comparatively reduced expression levels, expressing ADAMTSL5 in prokaryotes can mitigate these issues. In the present investigation, ADAMTSL5 expression was successfully achieved utilizing E. coli. Successive experiments will aim to validate the immunogenicity of the expressed proteins and to harvest antibodies for clinical studies via immunizing animals with ADAMTSL5. Despite the potential limitations of the prokaryotic expression system in accurately replicating complex protein structures, this study achieved relatively consistent protein yield and concentration. Furthermore, it employed economical and simple technical methods and effectively combined ADAMTSL5 with magnetic bead particles, providing designs and techniques for further studies on diagnosing psoriasis and related disorders.

## Author Contributions

**Ru Qin:** data curation; validation, writing–original draft, writing–review and editing. **Shangyang Li:** formal analysis, visualization. **Boheng Wu:** investigation. **Ruilan Lin:** investigation. **Yulin Yuan:** methodology, project administration, supervision, writing–review and editing.

## Ethics Statement

This study was approved by the ethics committee of The People's Hospital of Guangxi Zhuang Autonomous Region (Ethics No. KY‐KJT‐2021‐83). Informed written consents were obtained from all participants prior to before the commencement of the study.

## Conflicts of Interest

The authors declare no conflicts of interest.

## Data Availability

The data that support the findings of this study are available from the corresponding author upon reasonable request.
